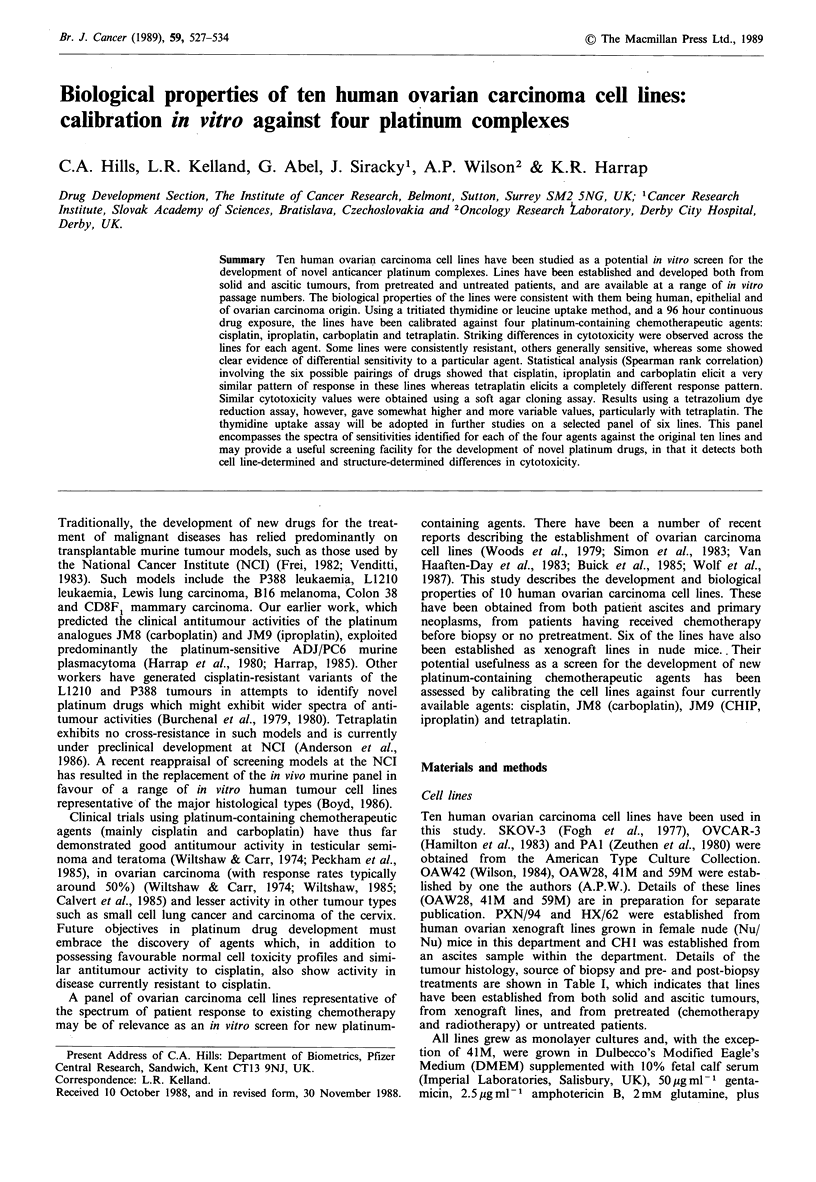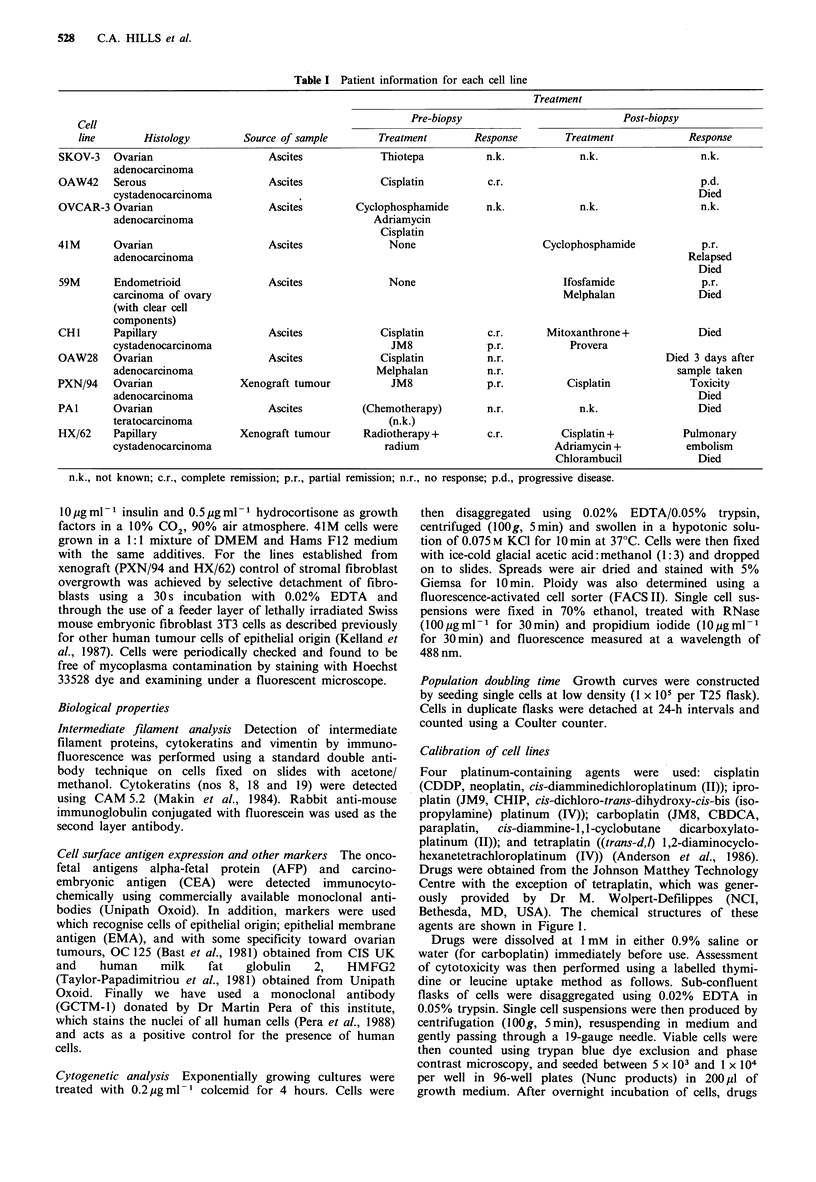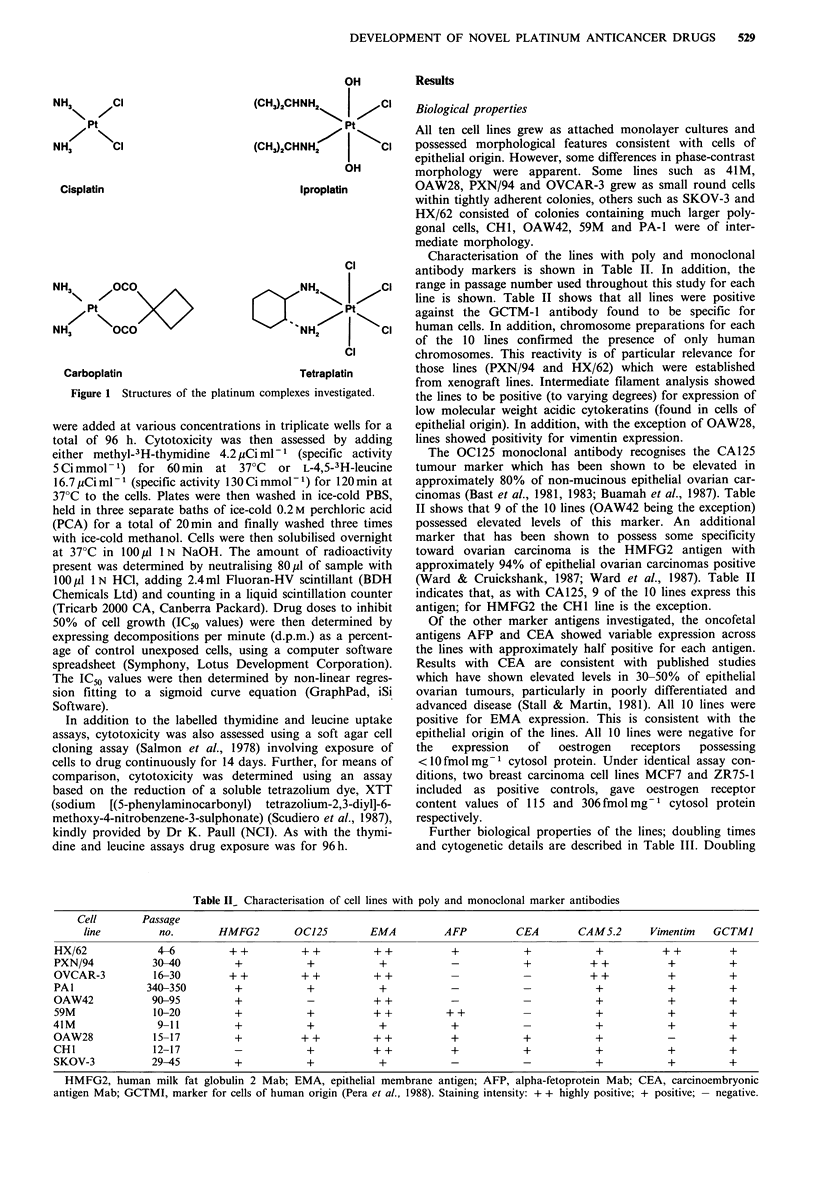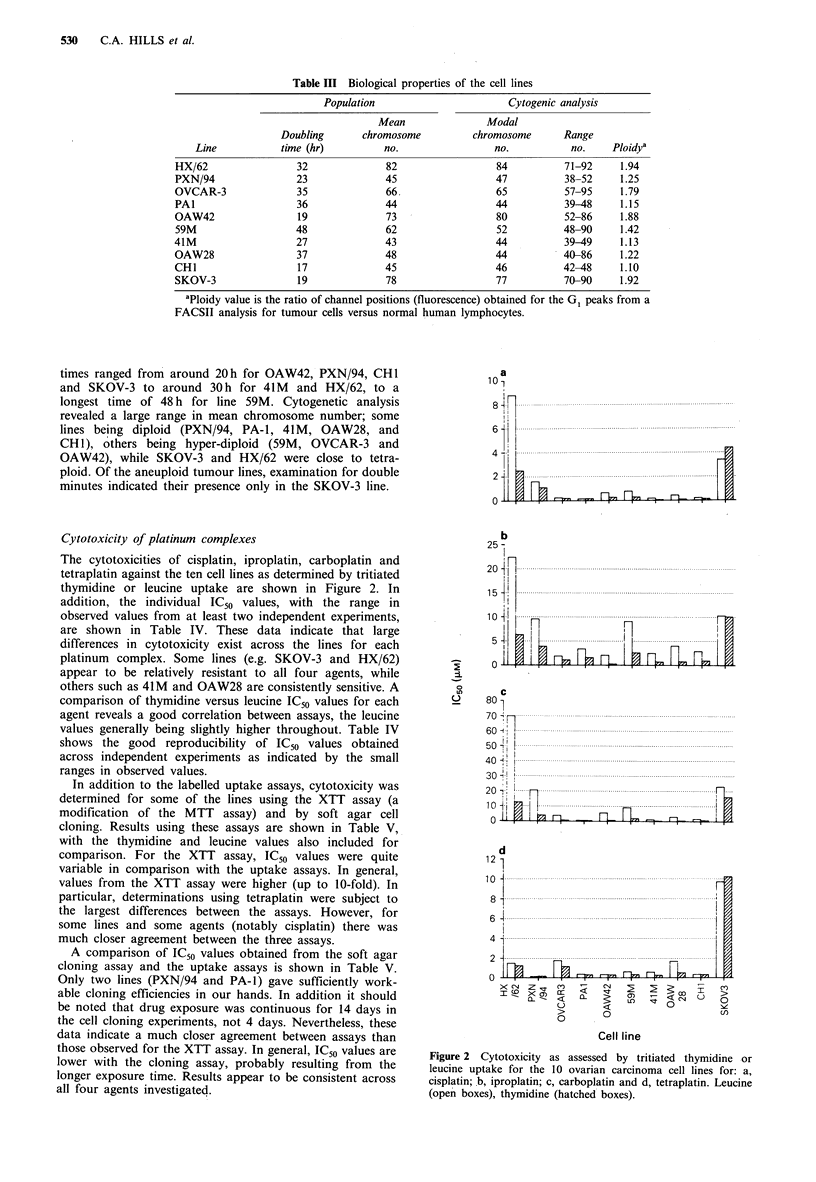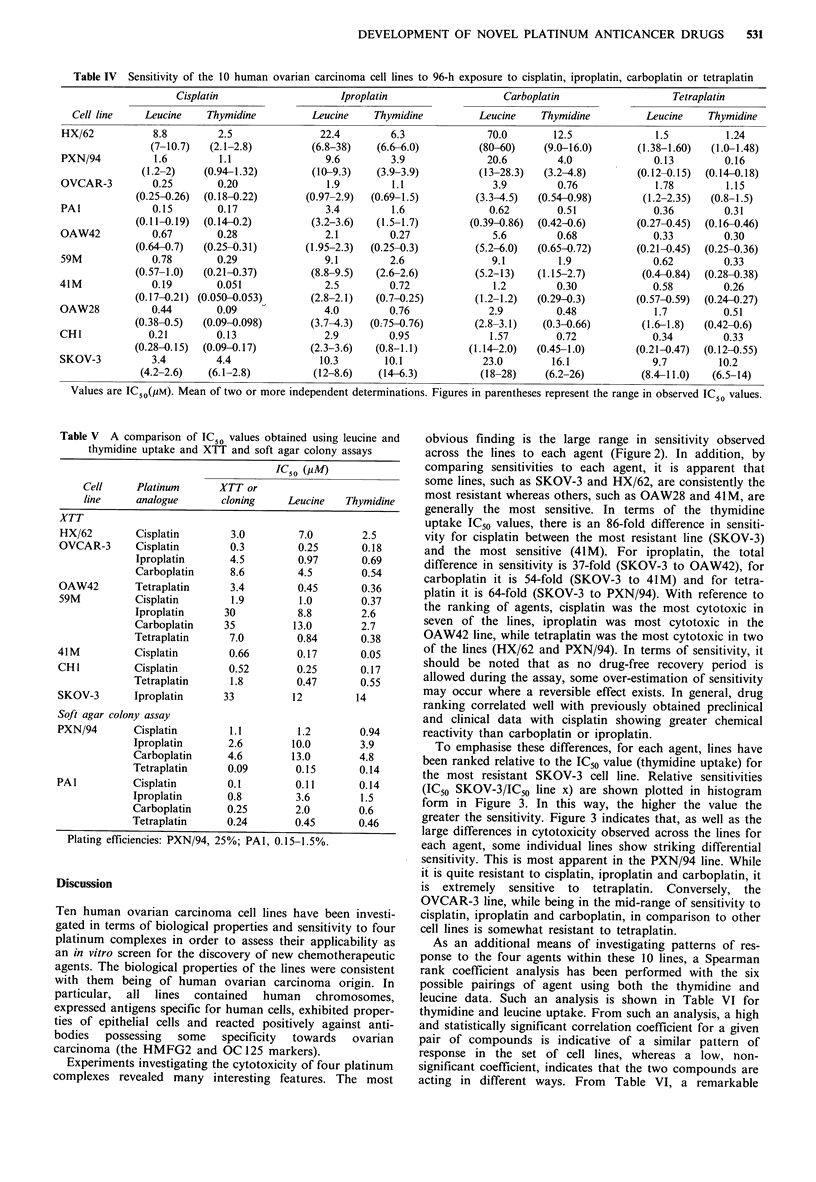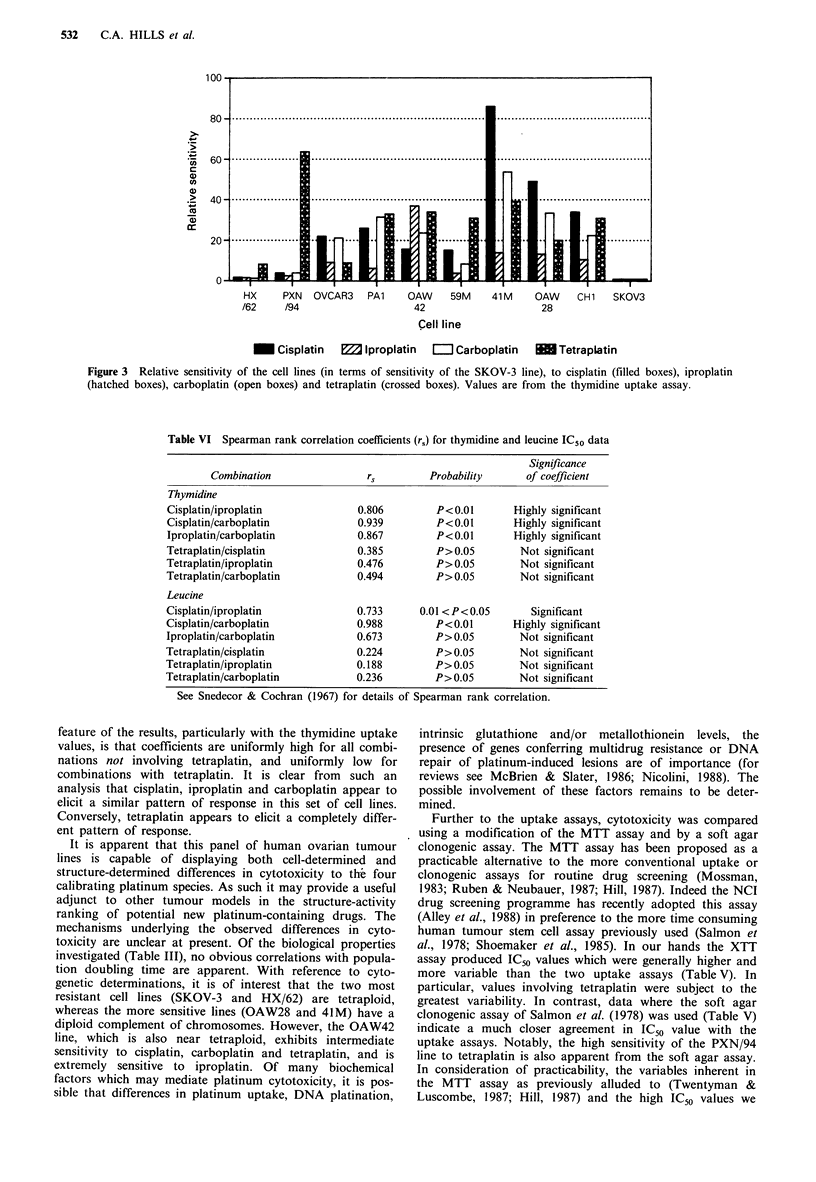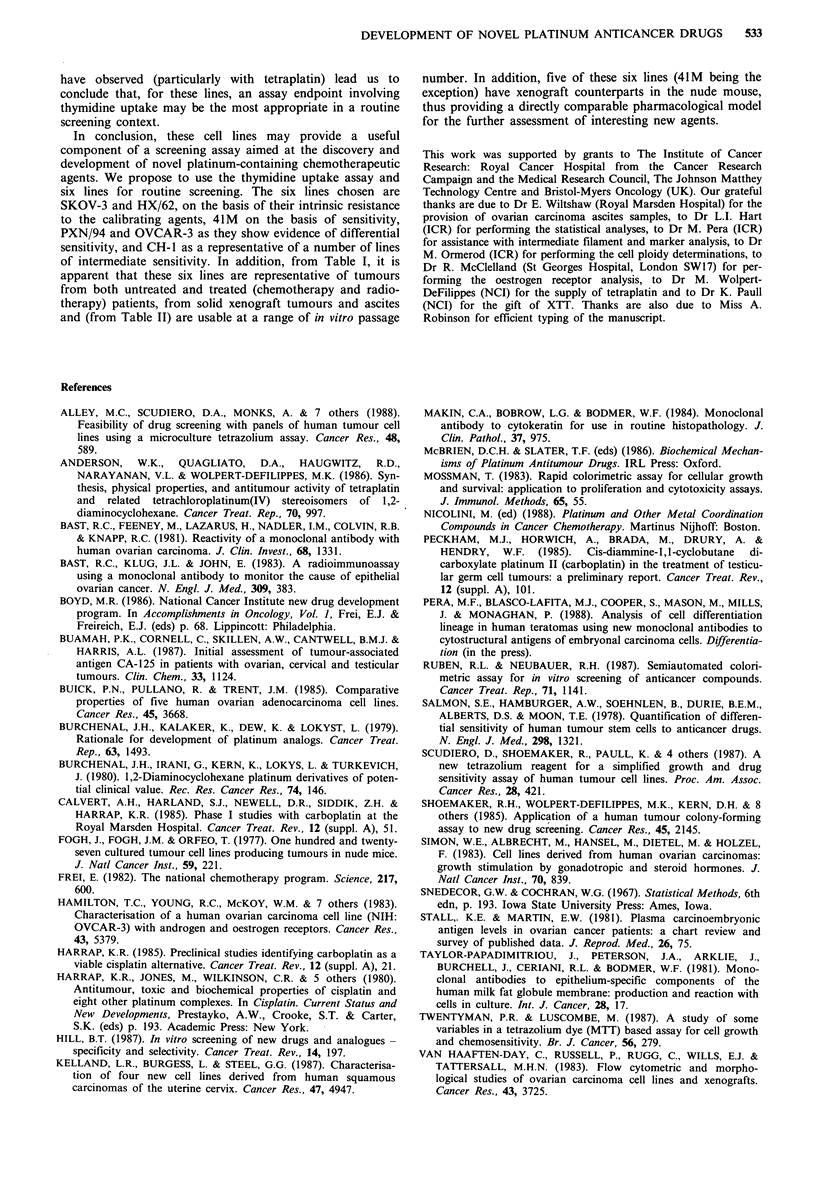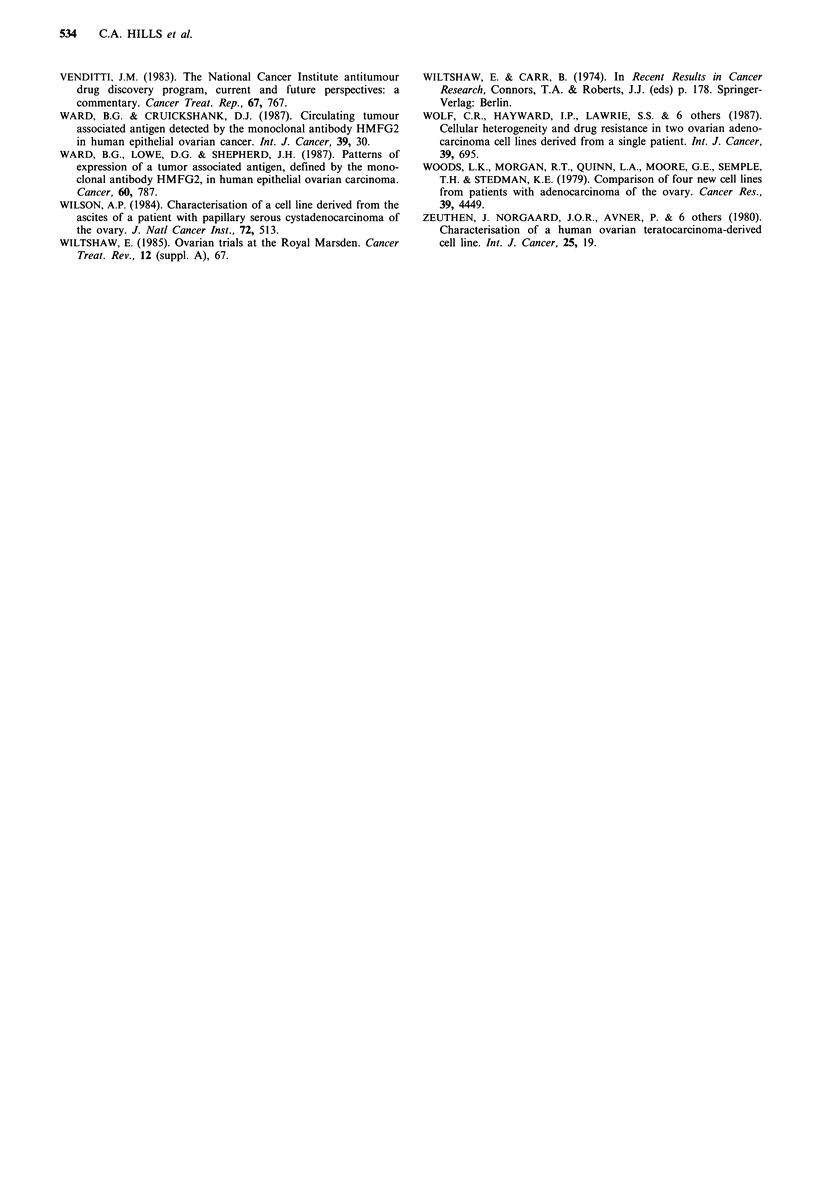# Biological properties of ten human ovarian carcinoma cell lines: calibration in vitro against four platinum complexes.

**DOI:** 10.1038/bjc.1989.108

**Published:** 1989-04

**Authors:** C. A. Hills, L. R. Kelland, G. Abel, J. Siracky, A. P. Wilson, K. R. Harrap

**Affiliations:** Drug Development Section, Institute of Cancer Research, Belmont, Sutton, Surrey, UK.

## Abstract

Ten human ovarian carcinoma cell lines have been studied as a potential in vitro screen for the development of novel anticancer platinum complexes. Lines have been established and developed both from solid and ascitic tumours, from pretreated and untreated patients, and are available at a range of in vitro passage numbers. The biological properties of the lines were consistent with them being human, epithelial and of ovarian carcinoma origin. Using a tritiated thymidine or leucine uptake method, and a 96 hour continuous drug exposure, the lines have been calibrated against four platinum-containing chemotherapeutic agents: cisplatin, iproplatin, carboplatin and tetraplatin. Striking differences in cytotoxicity were observed across the lines for each agent. Some lines were consistently resistant, others generally sensitive, whereas some showed clear evidence of differential sensitivity to a particular agent. Statistical analysis (Spearman rank correlation) involving the six possible pairings of drugs showed that cisplatin, iproplatin and carboplatin elicit a very similar pattern of response in these lines whereas tetraplatin elicits a completely different response pattern. Similar cytotoxicity values were obtained using a soft agar cloning assay. Results using a tetrazolium dye reduction assay, however, gave somewhat higher and more variable values, particularly with tetraplatin. The thymidine uptake assay will be adopted in further studies on a selected panel of six lines. This panel encompasses the spectra of sensitivities identified for each of the four agents against the original ten lines and may provide a useful screening facility for the development of novel platinum drugs, in that it detects both cell line-determined and structure-determined differences in cytotoxicity.


Br. J. Cancer (1989), 59, 527–534 © The Macmillan Press Ltd., 1989

### Biological properties of ten human ovarian carcinoma cell lines: calibration *in vitro* against four platinum complexes

C.A. Hills, L.R. Kelland, G. Abel, J. Siracky[1], A.P. Wilson[2] & K.R. Harrap

*Drug Development Section, The Institute of Cancer Research, Belmont, Sutton, Surrey SM2 5NG, UK; [1]Cancer Research Institute, Slovak Academy of Sciences, Bratislava, Czechoslovakia and [2]Oncology Research Laboratory, Derby City Hospital, Derby, UK.*

**Summary** Ten human ovarian carcinoma cell lines have been studied as a potential *in vitro* screen for the development of novel anticancer platinum complexes. Lines have been established and developed both from solid and ascitic tumours, from pretreated and untreated patients, and are available at a range of *in vitro* passage numbers. The biological properties of the lines were consistent with them being human, epithelial and of ovarian carcinoma origin. Using a tritiated thymidine or leucine uptake method, and a 96 hour continuous drug exposure, the lines have been calibrated against four platinum-containing chemotherapeutic agents: cisplatin, iproplatin, carboplatin and tetraplatin. Striking differences in cytotoxicity were observed across the lines for each agent. Some lines were consistently resistant, others generally sensitive, whereas some showed clear evidence of differential sensitivity to a particular agent. Statistical analysis (Spearman rank correlation) involving the six possible pairings of drugs showed that cisplatin, iproplatin and carboplatin elicit a very similar pattern of response in these lines whereas tetraplatin elicits a completely different response pattern. Similar cytotoxicity values were obtained using a soft agar cloning assay. Results using a tetrazolium dye reduction assay, however, gave somewhat higher and more variable values, particularly with tetraplatin. The thymidine uptake assay will be adopted in further studies on a selected panel of six lines. This panel encompasses the spectra of sensitivities identified for each of the four agents against the original ten lines and may provide a useful screening facility for the development of novel platinum drugs, in that it detects both cell line-determined and structure-determined differences in cytotoxicity.

Traditionally, the development of new drugs for the treatment of malignant diseases has relied predominantly on transplantable murine tumour models, such as those used by the National Cancer Institute (NCI) (Frei, 1982; Venditti, 1983). Such models include the P388 leukaemia, L1210 leukaemia, Lewis lung carcinoma, B16 melanoma, Colon 38 and CD8$F_1$ mammary carcinoma. Our earlier work, which predicted the clinical antitumour activities of the platinum analogues JM8 (carboplatin) and JM9 (iproplatin), exploited predominantly the platinum-sensitive ADJ/PC6 murine plasmacytoma (Harrap *et al.*, 1980; Harrap, 1985). Other workers have generated cisplatin-resistant variants of the L1210 and P388 tumours in attempts to identify novel platinum drugs which might exhibit wider spectra of antitumour activities (Burchenal *et al.*, 1979, 1980). Tetraplatin exhibits no cross-resistance in such models and is currently under preclinical development at NCI (Anderson *et al.*, 1986). A recent reappraisal of screening models at the NCI has resulted in the replacement of the *in vivo* murine panel in favour of a range of *in vitro* human tumour cell lines representative of the major histological types (Boyd, 1986).

Clinical trials using platinum-containing chemotherapeutic agents (mainly cisplatin and carboplatin) have thus far demonstrated good antitumour activity in testicular seminoma and teratoma (Wiltshaw & Carr, 1974; Peckham *et al.*, 1985), in ovarian carcinoma (with response rates typically around 50%) (Wiltshaw & Carr, 1974; Wiltshaw, 1985; Calvert *et al.*, 1985) and lesser activity in other tumour types such as small cell lung cancer and carcinoma of the cervix. Future objectives in platinum drug development must embrace the discovery of agents which, in addition to possessing favourable normal cell toxicity profiles and similar antitumour activity to cisplatin, also show activity in disease currently resistant to cisplatin.

A panel of ovarian carcinoma cell lines representative of the spectrum of patient response to existing chemotherapy may be of relevance as an *in vitro* screen for new platinum-containing agents. There have been a number of recent reports describing the establishment of ovarian carcinoma cell lines (Woods *et al.*, 1979; Simon *et al.*, 1983; Van Haaften-Day *et al.*, 1983; Buick *et al.*, 1985; Wolf *et al.*, 1987). This study describes the development and biological properties of 10 human ovarian carcinoma cell lines. These have been obtained from both patient ascites and primary neoplasms, from patients having received chemotherapy before biopsy or no pretreatment. Six of the lines have also been established as xenograft lines in nude mice. Their potential usefulness as a screen for the development of new platinum-containing chemotherapeutic agents has been assessed by calibrating the cell lines against four currently available agents: cisplatin, JM8 (carboplatin), JM9 (CHIP, iproplatin) and tetraplatin.

### Materials and methods

### *Cell lines*

Ten human ovarian carcinoma cell lines have been used in this study. SKOV-3 (Fogh *et al.*, 1977), OVCAR-3 (Hamilton *et al.*, 1983) and PA1 (Zeuthen *et al.*, 1980) were obtained from the American Type Culture Collection. OAW42 (Wilson, 1984), OAW28, 41M and 59M were established by one the authors (A.P.W.). Details of these lines (OAW28, 41M and 59M) are in preparation for separate publication. PXN/94 and HX/62 were established from human ovarian xenograft lines grown in female nude (Nu/Nu) mice in this department and CH1 was established from an ascites sample within the department. Details of the tumour histology, source of biopsy and pre- and post-biopsy treatments are shown in Table I, which indicates that lines have been established from both solid and ascitic tumours, from xenograft lines, and from pretreated (chemotherapy and radiotherapy) or untreated patients.

All lines grew as monolayer cultures and, with the exception of 41M, were grown in Dulbecco's Modified Eagle's Medium (DMEM) supplemented with 10% fetal calf serum (Imperial Laboratories, Salisbury, UK), 50 $\mu g\,ml^{-1}$ gentamicin, 2.5 $\mu g\,ml^{-1}$ amphotericin B, 2 mM glutamine, plus

Present Address of C.A. Hills: Department of Biometrics, Pfizer Central Research, Sandwich, Kent CT13 9NJ, UK.
Correspondence: L.R. Kelland.
Received 10 October 1988, and in revised form, 30 November 1988.

**Table I** Patient information for each cell line

| Cell line | Histology | Source of sample | Treatment: Pre-biopsy Treatment | Treatment: Pre-biopsy Response | Treatment: Post-biopsy Treatment | Treatment: Post-biopsy Response |
|---|---|---|---|---|---|---|
| SKOV-3 | Ovarian adenocarcinoma | Ascites | Thiotepa | n.k. | n.k. | n.k. |
| OAW42 | Serous cystadenocarcinoma | Ascites | Cisplatin | c.r. | | p.d. Died |
| OVCAR-3 | Ovarian adenocarcinoma | Ascites | Cyclophosphamide Adriamycin Cisplatin | n.k. | n.k. | n.k. |
| 41M | Ovarian adenocarcinoma | Ascites | None | | Cyclophosphamide | p.r. Relapsed Died |
| 59M | Endometrioid carcinoma of ovary (with clear cell components) | Ascites | None | | Ifosfamide Melphalan | p.r. Died |
| CH1 | Papillary cystadenocarcinoma | Ascites | Cisplatin JM8 | c.r. p.r. | Mitoxanthrone + Provera | Died |
| OAW28 | Ovarian adenocarcinoma | Ascites | Cisplatin Melphalan | n.r. n.r. | | Died 3 days after sample taken |
| PXN/94 | Ovarian adenocarcinoma | Xenograft tumour | JM8 | p.r. | Cisplatin | Toxicity Died |
| PA1 | Ovarian teratocarcinoma | Ascites | (Chemotherapy) (n.k.) | n.r. | n.k. | Died |
| HX/62 | Papillary cystadenocarcinoma | Xenograft tumour | Radiotherapy + radium | c.r. | Cisplatin + Adriamycin + Chlorambucil | Pulmonary embolism Died |

n.k., not known; c.r., complete remission; p.r., partial remission; n.r., no response; p.d., progressive disease.

10 $\mu g ml^{-1}$ insulin and 0.5 $\mu g ml^{-1}$ hydrocortisone as growth factors in a 10% $CO_2$, 90% air atmosphere. 41M cells were grown in a 1:1 mixture of DMEM and Hams F12 medium with the same additives. For the lines established from xenograft (PXN/94 and HX/62) control of stromal fibroblast overgrowth was achieved by selective detachment of fibroblasts using a 30 s incubation with 0.02% EDTA and through the use of a feeder layer of lethally irradiated Swiss mouse embryonic fibroblast 3T3 cells as described previously for other human tumour cells of epithelial origin (Kelland *et al.*, 1987). Cells were periodically checked and found to be free of mycoplasma contamination by staining with Hoechst 33528 dye and examining under a fluorescent microscope.

*Biological properties*

*Intermediate filament analysis* Detection of intermediate filament proteins, cytokeratins and vimentin by immunofluorescence was performed using a standard double antibody technique on cells fixed on slides with acetone/methanol. Cytokeratins (nos 8, 18 and 19) were detected using CAM 5.2 (Makin *et al.*, 1984). Rabbit anti-mouse immunoglobulin conjugated with fluorescein was used as the second layer antibody.

*Cell surface antigen expression and other markers* The oncofetal antigens alpha-fetal protein (AFP) and carcinoembryonic antigen (CEA) were detected immunocytochemically using commercially available monoclonal antibodies (Unipath Oxoid). In addition, markers were used which recognise cells of epithelial origin; epithelial membrane antigen (EMA), and with some specificity toward ovarian tumours, OC 125 (Bast *et al.*, 1981) obtained from CIS UK and human milk fat globulin 2, HMFG2 (Taylor-Papadimitriou *et al.*, 1981) obtained from Unipath Oxoid. Finally we have used a monoclonal antibody (GCTM-1) donated by Dr Martin Pera of this institute, which stains the nuclei of all human cells (Pera *et al.*, 1988) and acts as a positive control for the presence of human cells.

*Cytogenetic analysis* Exponentially growing cultures were treated with 0.2 $\mu g ml^{-1}$ colcemid for 4 hours. Cells were then disaggregated using 0.02% EDTA/0.05% trypsin, centrifuged (100 *g*, 5 min) and swollen in a hypotonic solution of 0.075 M KCl for 10 min at 37°C. Cells were then fixed with ice-cold glacial acetic acid: methanol (1:3) and dropped on to slides. Spreads were air dried and stained with 5% Giemsa for 10 min. Ploidy was also determined using a fluorescence-activated cell sorter (FACS II). Single cell suspensions were fixed in 70% ethanol, treated with RNase (100 $\mu g ml^{-1}$ for 30 min) and propidium iodide (10 $\mu g ml^{-1}$ for 30 min) and fluorescence measured at a wavelength of 488 nm.

*Population doubling time* Growth curves were constructed by seeding single cells at low density ($1 \times 10^5$ per T25 flask). Cells in duplicate flasks were detached at 24-h intervals and counted using a Coulter counter.

*Calibration of cell lines*

Four platinum-containing agents were used: cisplatin (CDDP, neoplatin, *cis*-diamminedichloroplatinum (II)); iproplatin (JM9, CHIP, *cis*-dichloro-*trans*-dihydroxy-*cis*-bis (isopropylamine) platinum (IV)); carboplatin (JM8, CBDCA, paraplatin, *cis*-diammine-1,1-cyclobutane dicarboxylatoplatinum (II)); and tetraplatin ((*trans-d,l*) 1,2-diaminocyclohexanetetrachloroplatinum (IV)) (Anderson *et al.*, 1986). Drugs were obtained from the Johnson Matthey Technology Centre with the exception of tetraplatin, which was generously provided by Dr M. Wolpert-Defilippes (NCI, Bethesda, MD, USA). The chemical structures of these agents are shown in Figure 1.

Drugs were dissolved at 1 mM in either 0.9% saline or water (for carboplatin) immediately before use. Assessment of cytotoxicity was then performed using a labelled thymidine or leucine uptake method as follows. Sub-confluent flasks of cells were disaggregated using 0.02% EDTA in 0.05% trypsin. Single cell suspensions were then produced by centrifugation (100 *g*, 5 min), resuspending in medium and gently passing through a 19-gauge needle. Viable cells were then counted using trypan blue dye exclusion and phase contrast microscopy, and seeded between $5 \times 10^3$ and $1 \times 10^4$ per well in 96-well plates (Nunc products) in 200 $\mu l$ of growth medium. After overnight incubation of cells, drugs

**Figure 1** Structures of the platinum complexes investigated.

were added at various concentrations in triplicate wells for a total of 96 h. Cytotoxicity was then assessed by adding either methyl-$^3$H-thymidine 4.2 $\mu$Ci ml$^{-1}$ (specific activity 5 Ci mmol$^{-1}$) for 60 min at 37°C or L-4,5-$^3$H-leucine 16.7 $\mu$Ci ml$^{-1}$ (specific activity 130 Ci mmol$^{-1}$) for 120 min at 37°C to the cells. Plates were then washed in ice-cold PBS, held in three separate baths of ice-cold 0.2 M perchloric acid (PCA) for a total of 20 min and finally washed three times with ice-cold methanol. Cells were then solubilised overnight at 37°C in 100 $\mu$l 1 N NaOH. The amount of radioactivity present was determined by neutralising 80 $\mu$l of sample with 100 $\mu$l 1 N HCl, adding 2.4 ml Fluoran-HV scintillant (BDH Chemicals Ltd) and counting in a liquid scintillation counter (Tricarb 2000 CA, Canberra Packard). Drug doses to inhibit 50% of cell growth ($IC_{50}$ values) were then determined by expressing decompositions per minute (d.p.m.) as a percentage of control unexposed cells, using a computer software spreadsheet (Symphony, Lotus Development Corporation). The $IC_{50}$ values were then determined by non-linear regression fitting to a sigmoid curve equation (GraphPad, iSi Software).

In addition to the labelled thymidine and leucine uptake assays, cytotoxicity was also assessed using a soft agar cell cloning assay (Salmon *et al.*, 1978) involving exposure of cells to drug continuously for 14 days. Further, for means of comparison, cytotoxicity was determined using an assay based on the reduction of a soluble tetrazolium dye, XTT (sodium [(5-phenylaminocarbonyl) tetrazolium-2,3-diyl]-6-methoxy-4-nitrobenzene-3-sulphonate) (Scudiero *et al.*, 1987), kindly provided by Dr K. Paull (NCI). As with the thymidine and leucine assays drug exposure was for 96 h.

### Results

### *Biological properties*

All ten cell lines grew as attached monolayer cultures and possessed morphological features consistent with cells of epithelial origin. However, some differences in phase-contrast morphology were apparent. Some lines such as 41M, OAW28, PXN/94 and OVCAR-3 grew as small round cells within tightly adherent colonies, others such as SKOV-3 and HX/62 consisted of colonies containing much larger polygonal cells, CH1, OAW42, 59M and PA-1 were of intermediate morphology.

Characterisation of the lines with poly and monoclonal antibody markers is shown in Table II. In addition, the range in passage number used throughout this study for each line is shown. Table II shows that all lines were positive against the GCTM-1 antibody found to be specific for human cells. In addition, chromosome preparations for each of the 10 lines confirmed the presence of only human chromosomes. This reactivity is of particular relevance for those lines (PXN/94 and HX/62) which were established from xenograft lines. Intermediate filament analysis showed the lines to be positive (to varying degrees) for expression of low molecular weight acidic cytokeratins (found in cells of epithelial origin). In addition, with the exception of OAW28, lines showed positivity for vimentin expression.

The OC125 monoclonal antibody recognises the CA125 tumour marker which has been shown to be elevated in approximately 80% of non-mucinous epithelial ovarian carcinomas (Bast *et al.*, 1981, 1983; Buamah *et al.*, 1987). Table II shows that 9 of the 10 lines (OAW42 being the exception) possessed elevated levels of this marker. An additional marker that has been shown to possess some specificity toward ovarian carcinoma is the HMFG2 antigen with approximately 94% of epithelial ovarian carcinomas positive (Ward & Cruickshank, 1987; Ward *et al.*, 1987). Table II indicates that, as with CA125, 9 of the 10 lines express this antigen; for HMFG2 the CH1 line is the exception.

Of the other marker antigens investigated, the oncofetal antigens AFP and CEA showed variable expression across the lines with approximately half positive for each antigen. Results with CEA are consistent with published studies which have shown elevated levels in 30–50% of epithelial ovarian tumours, particularly in poorly differentiated and advanced disease (Stall & Martin, 1981). All 10 lines were positive for EMA expression. This is consistent with the epithelial origin of the lines. All 10 lines were negative for the expression of oestrogen receptors possessing $< 10$ fmol mg$^{-1}$ cytosol protein. Under identical assay conditions, two breast carcinoma cell lines MCF7 and ZR75-1 included as positive controls, gave oestrogen receptor content values of 115 and 306 fmol mg$^{-1}$ cytosol protein respectively.

Further biological properties of the lines; doubling times and cytogenetic details are described in Table III. Doubling

**Table II** Characterisation of cell lines with poly and monoclonal marker antibodies

| *Cell line* | *Passage no.* | *HMFG2* | *OC125* | *EMA* | *AFP* | *CEA* | *CAM 5.2* | *Vimentim* | *GCTM1* |
|---|---|---|---|---|---|---|---|---|---|
| HX/62 | 4–6 | ++ | ++ | ++ | + | + | + | ++ | + |
| PXN/94 | 30–40 | + | + | + | – | + | ++ | + | + |
| OVCAR-3 | 16–30 | ++ | ++ | ++ | – | – | ++ | + | + |
| PA1 | 340–350 | + | + | + | – | – | + | + | + |
| OAW42 | 90–95 | + | – | ++ | – | – | + | + | + |
| 59M | 10–20 | + | + | ++ | ++ | – | + | + | + |
| 41M | 9–11 | + | + | + | + | – | + | + | + |
| OAW28 | 15–17 | + | ++ | ++ | + | + | + | – | + |
| CH1 | 12–17 | – | + | ++ | + | + | + | + | + |
| SKOV-3 | 29–45 | + | + | + | – | – | + | + | + |

HMFG2, human milk fat globulin 2 Mab; EMA, epithelial membrane antigen; AFP, alpha-fetoprotein Mab; CEA, carcinoembryonic antigen Mab; GCTMI, marker for cells of human origin (Pera *et al.*, 1988). Staining intensity: ++ highly positive; + positive; – negative.

**Table III** Biological properties of the cell lines

| Line | *Population* Doubling time (hr) | Mean chromosome no. | *Cytogenic analysis* Modal chromosome no. | Range no. | Ploidy[a] |
|---|---|---|---|---|---|
| HX/62 | 32 | 82 | 84 | 71–92 | 1.94 |
| PXN/94 | 23 | 45 | 47 | 38–52 | 1.25 |
| OVCAR-3 | 35 | 66. | 65 | 57–95 | 1.79 |
| PA1 | 36 | 44 | 44 | 39–48 | 1.15 |
| OAW42 | 19 | 73 | 80 | 52–86 | 1.88 |
| 59M | 48 | 62 | 52 | 48–90 | 1.42 |
| 41M | 27 | 43 | 44 | 39–49 | 1.13 |
| OAW28 | 37 | 48 | 44 | 40–86 | 1.22 |
| CH1 | 17 | 45 | 46 | 42–48 | 1.10 |
| SKOV-3 | 19 | 78 | 77 | 70–90 | 1.92 |

[a]Ploidy value is the ratio of channel positions (fluorescence) obtained for the $G_1$ peaks from a FACSII analysis for tumour cells versus normal human lymphocytes.

times ranged from around 20 h for OAW42, PXN/94, CH1 and SKOV-3 to around 30 h for 41M and HX/62, to a longest time of 48 h for line 59M. Cytogenetic analysis revealed a large range in mean chromosome number; some lines being diploid (PXN/94, PA-1, 41M, OAW28, and CH1), others being hyper-diploid (59M, OVCAR-3 and OAW42), while SKOV-3 and HX/62 were close to tetraploid. Of the aneuploid tumour lines, examination for double minutes indicated their presence only in the SKOV-3 line.

*Cytotoxicity of platinum complexes*

The cytotoxicities of cisplatin, iproplatin, carboplatin and tetraplatin against the ten cell lines as determined by tritiated thymidine or leucine uptake are shown in Figure 2. In addition, the individual $IC_{50}$ values, with the range in observed values from at least two independent experiments, are shown in Table IV. These data indicate that large differences in cytotoxicity exist across the lines for each platinum complex. Some lines (e.g. SKOV-3 and HX/62) appear to be relatively resistant to all four agents, while others such as 41M and OAW28 are consistently sensitive. A comparison of thymidine versus leucine $IC_{50}$ values for each agent reveals a good correlation between assays, the leucine values generally being slightly higher throughout. Table IV shows the good reproducibility of $IC_{50}$ values obtained across independent experiments as indicated by the small ranges in observed values.

In addition to the labelled uptake assays, cytotoxicity was determined for some of the lines using the XTT assay (a modification of the MTT assay) and by soft agar cell cloning. Results using these assays are shown in Table V, with the thymidine and leucine values also included for comparison. For the XTT assay, $IC_{50}$ values were quite variable in comparison with the uptake assays. In general, values from the XTT assay were higher (up to 10-fold). In particular, determinations using tetraplatin were subject to the largest differences between the assays. However, for some lines and some agents (notably cisplatin) there was much closer agreement between the three assays.

A comparison of $IC_{50}$ values obtained from the soft agar cloning assay and the uptake assays is shown in Table V. Only two lines (PXN/94 and PA-1) gave sufficiently workable cloning efficiencies in our hands. In addition it should be noted that drug exposure was continuous for 14 days in the cell cloning experiments, not 4 days. Nevertheless, these data indicate a much closer agreement between assays than those observed for the XTT assay. In general, $IC_{50}$ values are lower with the cloning assay, probably resulting from the longer exposure time. Results appear to be consistent across all four agents investigated.

**Figure 2** Cytotoxicity as assessed by tritiated thymidine or leucine uptake for the 10 ovarian carcinoma cell lines for: a, cisplatin; b, iproplatin; c, carboplatin and d, tetraplatin. Leucine (open boxes), thymidine (hatched boxes).

**Table IV** Sensitivity of the 10 human ovarian carcinoma cell lines to 96-h exposure to cisplatin, iproplatin, carboplatin or tetraplatin

| | *Cisplatin* | | *Iproplatin* | | *Carboplatin* | | *Tetraplatin* | |
|---|---|---|---|---|---|---|---|---|
| *Cell line* | *Leucine* | *Thymidine* | *Leucine* | *Thymidine* | *Leucine* | *Thymidine* | *Leucine* | *Thymidine* |
| HX/62 | 8.8 (7–10.7) | 2.5 (2.1–2.8) | 22.4 (6.8–38) | 6.3 (6.6–6.0) | 70.0 (80–60) | 12.5 (9.0–16.0) | 1.5 (1.38–1.60) | 1.24 (1.0–1.48) |
| PXN/94 | 1.6 (1.2–2) | 1.1 (0.94–1.32) | 9.6 (10–9.3) | 3.9 (3.9–3.9) | 20.6 (13–28.3) | 4.0 (3.2–4.8) | 0.13 (0.12–0.15) | 0.16 (0.14–0.18) |
| OVCAR-3 | 0.25 (0.25–0.26) | 0.20 (0.18–0.22) | 1.9 (0.97–2.9) | 1.1 (0.69–1.5) | 3.9 (3.3–4.5) | 0.76 (0.54–0.98) | 1.78 (1.2–2.35) | 1.15 (0.8–1.5) |
| PA1 | 0.15 (0.11–0.19) | 0.17 (0.14–0.2) | 3.4 (3.2–3.6) | 1.6 (1.5–1.7) | 0.62 (0.39–0.86) | 0.51 (0.42–0.6) | 0.36 (0.27–0.45) | 0.31 (0.16–0.46) |
| OAW42 | 0.67 (0.64–0.7) | 0.28 (0.25–0.31) | 2.1 (1.95–2.3) | 0.27 (0.25–0.3) | 5.6 (5.2–6.0) | 0.68 (0.65–0.72) | 0.33 (0.21–0.45) | 0.30 (0.25–0.36) |
| 59M | 0.78 (0.57–1.0) | 0.29 (0.21–0.37) | 9.1 (8.8–9.5) | 2.6 (2.6–2.6) | 9.1 (5.2–13) | 1.9 (1.15–2.7) | 0.62 (0.4–0.84) | 0.33 (0.28–0.38) |
| 41M | 0.19 (0.17–0.21) | 0.051 (0.050–0.053) | 2.5 (2.8–2.1) | 0.72 (0.7–0.25) | 1.2 (1.2–1.2) | 0.30 (0.29–0.3) | 0.58 (0.57–0.59) | 0.26 (0.24–0.27) |
| OAW28 | 0.44 (0.38–0.5) | 0.09 (0.09–0.098) | 4.0 (3.7–4.3) | 0.76 (0:75–0.76) | 2.9 (2.8–3.1) | 0.48 (0.3–0.66) | 1.7 (1.6–1.8) | 0.51 (0.42–0.6) |
| CH1 | 0.21 (0.28–0.15) | 0.13 (0.09–0.17) | 2.9 (2.3–3.6) | 0.95 (0.8–1.1) | 1.57 (1.14–2.0) | 0.72 (0.45–1.0) | 0.34 (0.21–0.47) | 0.33 (0.12–0.55) |
| SKOV-3 | 3.4 (4.2–2.6) | 4.4 (6.1–2.8) | 10.3 (12–8.6) | 10.1 (14–6.3) | 23.0 (18–28) | 16.1 (6.2–26) | 9.7 (8.4–11.0) | 10.2 (6.5–14) |

Values are $IC_{50}$($\mu$M). Mean of two or more independent determinations. Figures in parentheses represent the range in observed $IC_{50}$ values.

**Table V** A comparison of $IC_{50}$ values obtained using leucine and thymidine uptake and XTT and soft agar colony assays

| | | $IC_{50}$ ($\mu M$) | | |
|---|---|---|---|---|
| *Cell line* | *Platinum analogue* | *XTT or cloning* | *Leucine* | *Thymidine* |
| *XTT* | | | | |
| HX/62 | Cisplatin | 3.0 | 7.0 | 2.5 |
| OVCAR-3 | Cisplatin | 0.3 | 0.25 | 0.18 |
| | Iproplatin | 4.5 | 0.97 | 0.69 |
| | Carboplatin | 8.6 | 4.5 | 0.54 |
| OAW42 | Tetraplatin | 3.4 | 0.45 | 0.36 |
| 59M | Cisplatin | 1.9 | 1.0 | 0.37 |
| | Iproplatin | 30 | 8.8 | 2.6 |
| | Carboplatin | 35 | 13.0 | 2.7 |
| | Tetraplatin | 7.0 | 0.84 | 0.38 |
| 41M | Cisplatin | 0.66 | 0.17 | 0.05 |
| CH1 | Cisplatin | 0.52 | 0.25 | 0.17 |
| | Tetraplatin | 1.8 | 0.47 | 0.55 |
| SKOV-3 | Iproplatin | 33 | 12 | 14 |
| *Soft agar colony assay* | | | | |
| PXN/94 | Cisplatin | 1.1 | 1.2 | 0.94 |
| | Iproplatin | 2.6 | 10.0 | 3.9 |
| | Carboplatin | 4.6 | 13.0 | 4.8 |
| | Tetraplatin | 0.09 | 0.15 | 0.14 |
| PA1 | Cisplatin | 0.1 | 0.11 | 0.14 |
| | Iproplatin | 0.8 | 3.6 | 1.5 |
| | Carboplatin | 0.25 | 2.0 | 0.6 |
| | Tetraplatin | 0.24 | 0.45 | 0.46 |

Plating efficiencies: PXN/94, 25%; PA1, 0.15–1.5%.

### Discussion

Ten human ovarian carcinoma cell lines have been investigated in terms of biological properties and sensitivity to four platinum complexes in order to assess their applicability as an *in vitro* screen for the discovery of new chemotherapeutic agents. The biological properties of the lines were consistent with them being of human ovarian carcinoma origin. In particular, all lines contained human chromosomes, expressed antigens specific for human cells, exhibited properties of epithelial cells and reacted positively against antibodies possessing some specificity towards ovarian carcinoma (the HMFG2 and OC125 markers).

Experiments investigating the cytotoxicity of four platinum complexes revealed many interesting features. The most obvious finding is the large range in sensitivity observed across the lines to each agent (Figure 2). In addition, by comparing sensitivities to each agent, it is apparent that some lines, such as SKOV-3 and HX/62, are consistently the most resistant whereas others, such as OAW28 and 41M, are generally the most sensitive. In terms of the thymidine uptake $IC_{50}$ values, there is an 86-fold difference in sensitivity for cisplatin between the most resistant line (SKOV-3) and the most sensitive (41M). For iproplatin, the total difference in sensitivity is 37-fold (SKOV-3 to OAW42), for carboplatin it is 54-fold (SKOV-3 to 41M) and for tetraplatin it is 64-fold (SKOV-3 to PXN/94). With reference to the ranking of agents, cisplatin was the most cytotoxic in seven of the lines, iproplatin was most cytotoxic in the OAW42 line, while tetraplatin was the most cytotoxic in two of the lines (HX/62 and PXN/94). In terms of sensitivity, it should be noted that as no drug-free recovery period is allowed during the assay, some over-estimation of sensitivity may occur where a reversible effect exists. In general, drug ranking correlated well with previously obtained preclinical and clinical data with cisplatin showing greater chemical reactivity than carboplatin or iproplatin.

To emphasise these differences, for each agent, lines have been ranked relative to the $IC_{50}$ value (thymidine uptake) for the most resistant SKOV-3 cell line. Relative sensitivities ($IC_{50}$ SKOV-3/$IC_{50}$ line x) are shown plotted in histogram form in Figure 3. In this way, the higher the value the greater the sensitivity. Figure 3 indicates that, as well as the large differences in cytotoxicity observed across the lines for each agent, some individual lines show striking differential sensitivity. This is most apparent in the PXN/94 line. While it is quite resistant to cisplatin, iproplatin and carboplatin, it is extremely sensitive to tetraplatin. Conversely, the OVCAR-3 line, while being in the mid-range of sensitivity to cisplatin, iproplatin and carboplatin, in comparison to other cell lines is somewhat resistant to tetraplatin.

As an additional means of investigating patterns of response to the four agents within these 10 lines, a Spearman rank coefficient analysis has been performed with the six possible pairings of agent using both the thymidine and leucine data. Such an analysis is shown in Table VI for thymidine and leucine uptake. From such an analysis, a high and statistically significant correlation coefficient for a given pair of compounds is indicative of a similar pattern of response in the set of cell lines, whereas a low, non-significant coefficient, indicates that the two compounds are acting in different ways. From Table VI, a remarkable

**Figure 3** Relative sensitivity of the cell lines (in terms of sensitivity of the SKOV-3 line), to cisplatin (filled boxes), iproplatin (hatched boxes), carboplatin (open boxes) and tetraplatin (crossed boxes). Values are from the thymidine uptake assay.

**Table VI** Spearman rank correlation coefficients ($r_s$) for thymidine and leucine $IC_{50}$ data

| *Combination* | $r_s$ | *Probability* | *Significance of coefficient* |
|---|---|---|---|
| *Thymidine* | | | |
| Cisplatin/iproplatin | 0.806 | $P<0.01$ | Highly significant |
| Cisplatin/carboplatin | 0.939 | $P<0.01$ | Highly significant |
| Iproplatin/carboplatin | 0.867 | $P<0.01$ | Highly significant |
| Tetraplatin/cisplatin | 0.385 | $P>0.05$ | Not significant |
| Tetraplatin/iproplatin | 0.476 | $P>0.05$ | Not significant |
| Tetraplatin/carboplatin | 0.494 | $P>0.05$ | Not significant |
| *Leucine* | | | |
| Cisplatin/iproplatin | 0.733 | $0.01<P<0.05$ | Significant |
| Cisplatin/carboplatin | 0.988 | $P<0.01$ | Highly significant |
| Iproplatin/carboplatin | 0.673 | $P>0.05$ | Not significant |
| Tetraplatin/cisplatin | 0.224 | $P>0.05$ | Not significant |
| Tetraplatin/iproplatin | 0.188 | $P>0.05$ | Not significant |
| Tetraplatin/carboplatin | 0.236 | $P>0.05$ | Not significant |

See Snedecor & Cochran (1967) for details of Spearman rank correlation.

feature of the results, particularly with the thymidine uptake values, is that coefficients are uniformly high for all combinations *not* involving tetraplatin, and uniformly low for combinations with tetraplatin. It is clear from such an analysis that cisplatin, iproplatin and carboplatin appear to elicit a similar pattern of response in this set of cell lines. Conversely, tetraplatin appears to elicit a completely different pattern of response.

It is apparent that this panel of human ovarian tumour lines is capable of displaying both cell-determined and structure-determined differences in cytotoxicity to the four calibrating platinum species. As such it may provide a useful adjunct to other tumour models in the structure-activity ranking of potential new platinum-containing drugs. The mechanisms underlying the observed differences in cytotoxicity are unclear at present. Of the biological properties investigated (Table III), no obvious correlations with population doubling time are apparent. With reference to cytogenetic determinations, it is of interest that the two most resistant cell lines (SKOV-3 and HX/62) are tetraploid, whereas the more sensitive lines (OAW28 and 41M) have a diploid complement of chromosomes. However, the OAW42 line, which is also near tetraploid, exhibits intermediate sensitivity to cisplatin, carboplatin and tetraplatin, and is extremely sensitive to iproplatin. Of many biochemical factors which may mediate platinum cytotoxicity, it is possible that differences in platinum uptake, DNA platination, intrinsic glutathione and/or metallothionein levels, the presence of genes conferring multidrug resistance or DNA repair of platinum-induced lesions are of importance (for reviews see McBrien & Slater, 1986; Nicolini, 1988). The possible involvement of these factors remains to be determined.

Further to the uptake assays, cytotoxicity was compared using a modification of the MTT assay and by a soft agar clonogenic assay. The MTT assay has been proposed as a practicable alternative to the more conventional uptake or clonogenic assays for routine drug screening (Mossman, 1983; Ruben & Neubauer, 1987; Hill, 1987). Indeed the NCI drug screening programme has recently adopted this assay (Alley *et al.*, 1988) in preference to the more time consuming human tumour stem cell assay previously used (Salmon *et al.*, 1978; Shoemaker *et al.*, 1985). In our hands the XTT assay produced $IC_{50}$ values which were generally higher and more variable than the two uptake assays (Table V). In particular, values involving tetraplatin were subject to the greatest variability. In contrast, data where the soft agar clonogenic assay of Salmon *et al.* (1978) was used (Table V) indicate a much closer agreement in $IC_{50}$ value with the uptake assays. Notably, the high sensitivity of the PXN/94 line to tetraplatin is also apparent from the soft agar assay. In consideration of practicability, the variables inherent in the MTT assay as previously alluded to (Twentyman & Luscombe, 1987; Hill, 1987) and the high $IC_{50}$ values we have observed (particularly with tetraplatin) lead us to conclude that, for these lines, an assay endpoint involving thymidine uptake may be the most appropriate in a routine screening context.

In conclusion, these cell lines may provide a useful component of a screening assay aimed at the discovery and development of novel platinum-containing chemotherapeutic agents. We propose to use the thymidine uptake assay and six lines for routine screening. The six lines chosen are SKOV-3 and HX/62, on the basis of their intrinsic resistance to the calibrating agents, 41M on the basis of sensitivity, PXN/94 and OVCAR-3 as they show evidence of differential sensitivity, and CH-1 as a representative of a number of lines of intermediate sensitivity. In addition, from Table I, it is apparent that these six lines are representative of tumours from both untreated and treated (chemotherapy and radiotherapy) patients, from solid xenograft tumours and ascites and (from Table II) are usable at a range of *in vitro* passage number. In addition, five of these six lines (41M being the exception) have xenograft counterparts in the nude mouse, thus providing a directly comparable pharmacological model for the further assessment of interesting new agents.

This work was supported by grants to The Institute of Cancer Research: Royal Cancer Hospital from the Cancer Research Campaign and the Medical Research Council, The Johnson Matthey Technology Centre and Bristol-Myers Oncology (UK). Our grateful thanks are due to Dr E. Wiltshaw (Royal Marsden Hospital) for the provision of ovarian carcinoma ascites samples, to Dr L.I. Hart (ICR) for performing the statistical analyses, to Dr M. Pera (ICR) for assistance with intermediate filament and marker analysis, to Dr M. Ormerod (ICR) for performing the cell ploidy determinations, to Dr R. McClelland (St Georges Hospital, London SW17) for performing the oestrogen receptor analysis, to Dr M. Wolpert-DeFilippes (NCI) for the supply of tetraplatin and to Dr K. Paull (NCI) for the gift of XTT. Thanks are also due to Miss A. Robinson for efficient typing of the manuscript.

### References

ALLEY, M.C., SCUDIERO, D.A., MONKS, A. & 7 others (1988). Feasibility of drug screening with panels of human tumour cell lines using a microculture tetrazolium assay. *Cancer Res.*, **48**, 589.

ANDERSON, W.K., QUAGLIATO, D.A., HAUGWITZ, R.D., NARAYANAN, V.L. & WOLPERT-DEFILIPPES, M.K. (1986). Synthesis, physical properties, and antitumour activity of tetraplatin and related tetrachloroplatinum(IV) stereoisomers of 1,2-diaminocyclohexane. *Cancer Treat. Rep.*, **70**, 997.

BAST, R.C., FEENEY, M., LAZARUS, H., NADLER, I.M., COLVIN, R.B. & KNAPP, R.C. (1981). Reactivity of a monoclonal antibody with human ovarian carcinoma. *J. Clin. Invest.*, **68**, 1331.

BAST, R.C., KLUG, J.L. & JOHN, E. (1983). A radioimmunoassay using a monoclonal antibody to monitor the cause of epithelial ovarian cancer. *N. Engl. J. Med.*, **309**, 383.

BOYD, M.R. (1986). National Cancer Institute new drug development program. In *Accomplishments in Oncology, Vol. 1*, Frei, E.J. & Freireich, E.J. (eds) p. 68. Lippincott: Philadelphia.

BUAMAH, P.K., CORNELL, C., SKILLEN, A.W., CANTWELL, B.M.J. & HARRIS, A.L. (1987). Initial assessment of tumour-associated antigen CA-125 in patients with ovarian, cervical and testicular tumours. *Clin. Chem.*, **33**, 1124.

BUICK, P.N., PULLANO, R. & TRENT, J.M. (1985). Comparative properties of five human ovarian adenocarcinoma cell lines. *Cancer Res.*, **45**, 3668.

BURCHENAL, J.H., KALAKER, K., DEW, K. & LOKYST, L. (1979). Rationale for development of platinum analogs. *Cancer Treat. Rep.*, **63**, 1493.

BURCHENAL, J.H., IRANI, G., KERN, K., LOKYS, L. & TURKEVICH, J. (1980). 1,2-Diaminocyclohexane platinum derivatives of potential clinical value. *Rec. Res. Cancer Res.*, **74**, 146.

CALVERT, A.H., HARLAND, S.J., NEWELL, D.R., SIDDIK, Z.H. & HARRAP, K.R. (1985). Phase I studies with carboplatin at the Royal Marsden Hospital. *Cancer Treat. Rev.*, **12** (suppl. A), 51.

FOGH, J., FOGH, J.M. & ORFEO, T. (1977). One hundred and twenty-seven cultured tumour cell lines producing tumours in nude mice. *J. Natl Cancer Inst.*, **59**, 221.

FREI, E. (1982). The national chemotherapy program. *Science*, **217**, 600.

HAMILTON, T.C., YOUNG, R.C., McKOY, W.M. & 7 others (1983). Characterisation of a human ovarian carcinoma cell line (NIH: OVCAR-3) with androgen and oestrogen receptors. *Cancer Res.*, **43**, 5379.

HARRAP, K.R. (1985). Preclinical studies identifying carboplatin as a viable cisplatin alternative. *Cancer Treat. Rev.*, **12** (suppl. A), 21.

HARRAP, K.R., JONES, M., WILKINSON, C.R. & 5 others (1980). Antitumour, toxic and biochemical properties of cisplatin and eight other platinum complexes. In *Cisplatin. Current Status and New Developments*, Prestayko, A.W., Crooke, S.T. & Carter, S.K. (eds) p. 193. Academic Press: New York.

HILL, B.T. (1987). *In vitro* screening of new drugs and analogues – specificity and selectivity. *Cancer Treat. Rev.*, **14**, 197.

KELLAND, L.R., BURGESS, L. & STEEL, G.G. (1987). Characterisation of four new cell lines derived from human squamous carcinomas of the uterine cervix. *Cancer Res.*, **47**, 4947.

MAKIN, C.A., BOBROW, L.G. & BODMER, W.F. (1984). Monoclonal antibody to cytokeratin for use in routine histopathology. *J. Clin. Pathol.*, **37**, 975.

McBRIEN, D.C.H. & SLATER, T.F. (eds) (1986). *Biochemical Mechanisms of Platinum Antitumour Drugs*. IRL Press: Oxford.

MOSSMAN, T. (1983). Rapid colorimetric assay for cellular growth and survival: application to proliferation and cytotoxicity assays. *J. Immunol. Methods*, **65**, 55.

NICOLINI, M. (ed) (1988). *Platinum and Other Metal Coordination Compounds in Cancer Chemotherapy*. Martinus Nijhoff: Boston.

PECKHAM, M.J., HORWICH, A., BRADA, M., DRURY, A. & HENDRY, W.F. (1985). Cis-diammine-1,1-cyclobutane dicarboxylate platinum II (carboplatin) in the treatment of testicular germ cell tumours: a preliminary report. *Cancer Treat. Rev.*, **12** (suppl. A), 101.

PERA, M.F., BLASCO-LAFITA, M.J., COOPER, S., MASON, M., MILLS, J. & MONAGHAN, P. (1988). Analysis of cell differentiation lineage in human teratomas using new monoclonal antibodies to cytostructural antigens of embryonal carcinoma cells. *Differentiation* (in the press).

RUBEN, R.L. & NEUBAUER, R.H. (1987). Semiautomated colorimetric assay for *in vitro* screening of anticancer compounds. *Cancer Treat. Rep.*, **71**, 1141.

SALMON, S.E., HAMBURGER, A.W., SOEHNLEN, B., DURIE, B.E.M., ALBERTS, D.S. & MOON, T.E. (1978). Quantification of differential sensitivity of human tumour stem cells to anticancer drugs. *N. Engl. J. Med.*, **298**, 1321.

SCUDIERO, D., SHOEMAKER, R., PAULL, K. & 4 others (1987). A new tetrazolium reagent for a simplified growth and drug sensitivity assay of human tumour cell lines. *Proc. Am. Assoc. Cancer Res.*, **28**, 421.

SHOEMAKER, R.H., WOLPERT-DEFILIPPES, M.K., KERN, D.H. & 8 others (1985). Application of a human tumour colony-forming assay to new drug screening. *Cancer Res.*, **45**, 2145.

SIMON, W.E., ALBRECHT, M., HANSEL, M., DIETEL, M. & HOLZEL, F. (1983). Cell lines derived from human ovarian carcinomas: growth stimulation by gonadotropic and steroid hormones. *J. Natl Cancer Inst.*, **70**, 839.

SNEDECOR, G.W. & COCHRAN, W.G. (1967). *Statistical Methods*, 6th edn, p. 193. Iowa State University Press: Ames, Iowa.

STALL,. K.E. & MARTIN, E.W. (1981). Plasma carcinoembryonic antigen levels in ovarian cancer patients: a chart review and survey of published data. *J. Reprod. Med.*, **26**, 75.

TAYLOR-PAPADIMITRIOU, J., PETERSON, J.A., ARKLIE, J., BURCHELL, J., CERIANI, R.L. & BODMER, W.F. (1981). Monoclonal antibodies to epithelium-specific components of the human milk fat globule membrane: production and reaction with cells in culture. *Int. J. Cancer*, **28**, 17.

TWENTYMAN, P.R. & LUSCOMBE, M. (1987). A study of some variables in a tetrazolium dye (MTT) based assay for cell growth and chemosensitivity. *Br. J. Cancer*, **56**, 279.

VAN HAAFTEN-DAY, C., RUSSELL, P., RUGG, C., WILLS, E.J. & TATTERSALL, M.H.N. (1983). Flow cytometric and morphological studies of ovarian carcinoma cell lines and xenografts. *Cancer Res.*, **43**, 3725.

VENDITTI, J.M. (1983). The National Cancer Institute antitumour drug discovery program, current and future perspectives: a commentary. *Cancer Treat. Rep.*, **67**, 767.

WARD, B.G. & CRUICKSHANK, D.J. (1987). Circulating tumour associated antigen detected by the monoclonal antibody HMFG2 in human epithelial ovarian cancer. *Int. J. Cancer*, **39**, 30.

WARD, B.G., LOWE, D.G. & SHEPHERD, J.H. (1987). Patterns of expression of a tumor associated antigen, defined by the monoclonal antibody HMFG2, in human epithelial ovarian carcinoma. *Cancer*, **60**, 787.

WILSON, A.P. (1984). Characterisation of a cell line derived from the ascites of a patient with papillary serous cystadenocarcinoma of the ovary. *J. Natl Cancer Inst.*, **72**, 513.

WILTSHAW, E. (1985). Ovarian trials at the Royal Marsden. *Cancer Treat. Rev.*, **12** (suppl. A), 67.

WILTSHAW, E. & CARR, B. (1974). In *Recent Results in Cancer Research*, Connors, T.A. & Roberts, J.J. (eds) p. 178. Springer-Verlag: Berlin.

WOLF, C.R., HAYWARD, I.P., LAWRIE, S.S. & 6 others (1987). Cellular heterogeneity and drug resistance in two ovarian adenocarcinoma cell lines derived from a single patient. *Int. J. Cancer*, **39**, 695.

WOODS, L.K., MORGAN, R.T., QUINN, L.A., MOORE, G.E., SEMPLE, T.H. & STEDMAN, K.E. (1979). Comparison of four new cell lines from patients with adenocarcinoma of the ovary. *Cancer Res.*, **39**, 4449.

ZEUTHEN, J. NORGAARD, J.O.R., AVNER, P. & 6 others (1980). Characterisation of a human ovarian teratocarcinoma-derived cell line. *Int. J. Cancer*, **25**, 19.